# Human Bocavirus 2 in Children, South Korea

**DOI:** 10.3201/eid1510.090337

**Published:** 2009-10

**Authors:** Tae-Hee Han, Ju-Young Chung, Eung-Soo Hwang

**Affiliations:** Inje University, Seoul, South Korea (T.-H. Han, J.-Y. Chung); Seoul National University, Seoul (E.-S. Hwang)

**Keywords:** human bocavirus 2, children, acute lower respiratory tract infections, Kawasaki disease, Henoch-Schönlein purpura, viruses, letter

**To the Editor:** In 2009, Kapoor et al. and Arthur et al. published reports on the prevalence of the newly identified parvovirus, human bocavirus 2 (HBoV-2), in fecal samples (*1*,*2*). HBoV-1 had been discovered in 2005 (*3*), and reports indicate its possible role in respiratory diseases such as upper respiratory tract infections, lower respiratory tract infections (LRTIs), and in exacerbation of asthma (*4*); in these diseases, the virus co-infects with other respiratory viruses (*5*). Systemic infection with HBoV-1 and possible association of this virus with other diseases such as gastroenteritis, Kawasaki disease, and hepatitis have been reported (*6**–**8*). We looked for HBoV-2 in clinical samples from children with various diseases, including acute LRTIs, Kawasaki disease, Henoch-Schönlein purpura, and hepatitis.

During September 2008–January 2009, a total of 212 nasopharyngeal aspirates were collected from 212 children (median age 8 months, range 1–59 months) hospitalized with acute LRTIs at Sanggyepaik Hospital in Seoul, South Korea. Previously, during January 2002–June 2006, a total of 173 serum samples had been obtained from children (age range 1 month–15 years) with hepatitis (hepatitis B, 20 samples; hepatitis C, 11 samples; unknown hepatitis, 31 samples), Kawasaki disease (12 samples), and Henoch-Schönlein purpura (18 samples) and from healthy children (same age range, 81 samples) (*9*). The study was approved by the internal review board of Sanggyepaik Hospital.

DNA was extracted from serum samples, and RNA and DNA were extracted from nasopharyngeal aspirates by using a QIAamp Viral RNA Mini Kit (QIAGEN, Hilden, Germany) and a QIAamp DNA Blood Mini Kit (QIAGEN GmbH), respectively. All nasopharyngeal aspirates were tested by PCR for common respiratory viruses such as respiratory syncytial virus, influenza viruses A and B, parainfluenza virus, and adenovirus, as described previously (*10*). PCRs to detect HBoV-1 were performed by using primers for the nonstructural (NS) 1 and nucleocapsid protein (NP) 1 genes, as described previously (*10*). Additional PCRs for rhinovirus, human metapneumovirus, human coronavirus (hCoV)-NL63, hCoV-OC43, hCoV-229E, hCoV HKU-1, WU polyomavirus, and KU polyomavirus were performed, as described, for HBoV-2–positive samples (*10*).

HBoV-2 was detected by performing first-round PCR with primers based on the *NS* gene, HBoV2-sf1, and HBoV2-sr1. Second-round PCR was performed by using primers HBoV2-sf2 and HBoVsr2, as described previously (*1*). The PCR products were sequenced by using an ABI 3730 XL autoanalyzer (Applied Biosystems, Foster City, CA, USA). The nucleotide sequences were aligned by using BioEdit 7.0 (www.mbio.ncsu.edu/BioEdit/BioEdit.html) and presented in a topology tree, prepared by using MEGA 4.1 (www.megasoftware.net).

Of the 212 samples tested, the following viruses were detected: human respiratory syncytial virus (in 124 [58.4%] samples), human rhinovirus (24 [11.3%]), influenza virus A (18 [8.4%]), adenovirus (10 [4.7%]), and parainfluenza virus (8 [3.7%]). HBoV-1 was not detected in the study population. HBoV-2 DNA was found in 5 (2.3%) of the 212 samples collected; all positive samples had been obtained in October 2008. The age range of the children with HBoV-2–positive samples was 4–34 months (median 24 months), and all were male. The diagnoses were bronchiolitis for 3 children and bronchopneumonia for 2. The most frequently codetected virus was human respiratory syncytial virus, found in 4 (80%) of 5 samples. One sample that was negative for respiratory syncytial virus and positive for HBoV-2 was negative for all other respiratory viruses.

Nucleotide sequences were determined for the NS-1 gene, and phylogenetic analyses, which included HBoV-3, a new lineage designated by Arthur et al. (*2*), showed that the NS-1 gene was relatively well conserved and that there were 2 major groups of the virus, the UK strain and the Pakistan strain. HBoV-2 strains isolated from South Korea belonged to the HBoV-PK2255 (FJ170279) cluster (Figure).

**Figure:**
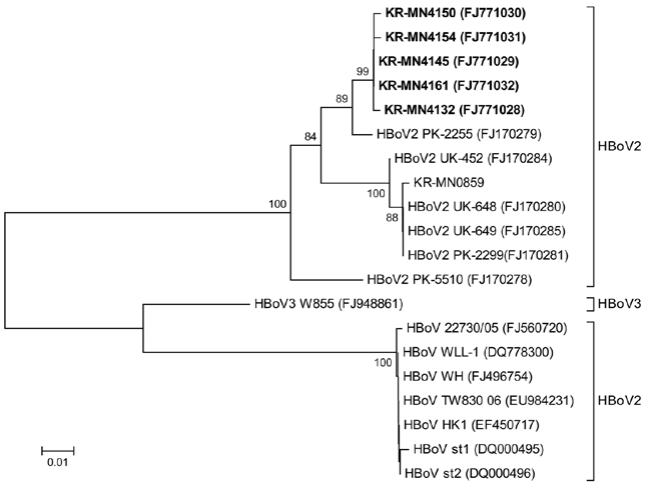
Phylogenetic analysis of nonstructural (NS) 1 gene sequences from human bocavirus 2 strains from Korea (KR), United Kingdom (UK), and Pakistan (PK), presented on a topology tree prepared by using MEGA 3.1 (www.megasoftware.net). Nucleotide alignment of a 417-bp portion of the NS1 gene was prepared by using BioEdit 7.0 (www.mbio.ncsu.edu/BioEdit/BioEdit.html). The nucleotide distance matrix was generated by using the Kimura 2-parameter estimation. Nodal confidence values indicate the results of bootstrap resampling (n = 1,000). Five strains from South Korea (FJ771028–32) are in **boldface.** Scale bar indicates estimated number of substitutions per 10 bases.

Recent studies have detected HBoV-1 in serum samples of children with Kawasaki disease and of an immunocompromised child with hepatitis (*7**,**8*). However, neither HBoV-1 nor HBoV-2 was detected in the 172 serum samples from 61 patients with hepatitis, 12 with Kawasaki disease, 18 with Henoch-Schönlein purpura, and 81 healthy children.

The absence of HBoV-1 in the samples examined was unexpected because HBoV-1 was detected in >10% of 558 respiratory samples collected from a demographically similar study population during the winter 2 years earlier (*10*). Future studies, with larger populations and over longer periods, are needed to delineate seasonal variations between HBoV-1 and HBoV-2.

We demonstrated HBoV-2 DNA in the respiratory tract secretions of children with acute LRTIs. In most positive samples, the virus was found in addition to other respiratory viruses. A limitation is that the study did not consider health control measures and other clinical disease such as gastroenteritis and was conducted for a short time. The role of HBoV-2 in LRTIs remains unclear; further studies are needed to clarify whether this virus is only shed from the respiratory tract or whether it replicates in the gastrointestinal tract.
